# Comparison of Growth and Composition of Black Soldier Fly (*Hermetia illucens* L.) Larvae Reared on Sugarcane By-Products and Other Substrates

**DOI:** 10.3390/insects15100771

**Published:** 2024-10-06

**Authors:** Nooshin Zandi-Sohani, Jeffery K. Tomberlin

**Affiliations:** 1 Department of Plant Protection, Faculty of Agriculture, Agricultural Sciences and Natural Resources University of Khuzestan, Mollasani 63417-73637, Iran; 2 Department of Entomology, Texas A&M University, College Station, TX 77840, USA; jeffery.tomberlin@ag.tamu.edu

**Keywords:** entomophagy, food waste, sugarcane by-product

## Abstract

**Simple Summary:**

The black soldier fly (BSF) has recently gained global popularity for converting organic waste into valuable biomass for animal feed. Previous studies have demonstrated the ability of BSF larvae to consume various food wastes and animal manure. In this study, we used sugarcane by-products—such as bagasse, vinasse, and molasses—as a food source for BSF larvae and compared their development and nutritional value with those fed with university canteen leftovers (UCLs). The BSF larvae grew longer and heavier when fed university canteen leftovers compared to other treatments. The UCL treatment also resulted in the larvae’s highest protein and oil content. Overall, the BSF larvae exhibited the best growth on the UCL substrate, while the combination of bagasse and vinasse (BV) proved to be the most suitable substrate among the sugarcane by-products for BSF larva development.

**Abstract:**

Black soldier fly larvae (BSFL) can convert organic waste into high-quality biomass. In this study, we tested the potential of sugarcane by-products as a food source for BSFL and compared larval development and nutritional value with some other organic substrates. Seven different substrates were used, including carrot pomace (C), carrot pomace and leftover bread (CB) (50/50), bagasse and vinasse (BV), bagasse and molasses (BM), bagasse, vinasse, and molasses (BVM), a mixture of all the above treatments (MX), and university canteen leftovers (UCLs). The larval weight and length were measured for two weeks from day 5 to 19. Then, the BSFL were harvested and analyzed for dry matter, crude protein, oil, ash, mineral, and fatty acid composition. Larval weight and length varied depending on the feeding substrate provided. University canteen leftovers resulted in the BSFL having at least 18% greater length (17.00 mm) and 56% greater weight (3.15 g) compared to other treatments. The highest amounts of protein (38.9%) and oil (39.06%) were observed in the UCL treatment, while the BV treatment larvae had the highest quantities of ash (28.9%) and dry matter (28.0%). The fatty acid profile of the BSFL revealed three-times-higher levels of saturated fatty acids than unsaturated fatty acids in the UCL treatment and was at least twice as high in other treatments. Overall, the BSFL had the best growth on the UCL substrate, and the combination of bagasse and vinasse (BV) was the most appropriate substrate for BSFL development among the sugarcane by-products.

## 1. Introduction

The global population is projected to reach 9.8 billion by 2050, with most of this growth occurring in Global South nations [1]. Therefore, to sustainably feed the world, the demand for food production should rise by 50% by 2030 and by 70–100% of current levels by 2050 [2].

One of the challenges faced by this demand for increased food production is the constraints on natural resources such as land, energy, and water. On the other hand, food production in its current method brings about issues such as deforestation, environmental degradation, and greenhouse gas emissions [3]. Therefore, it is important to identify new food sources that are less associated with the challenges mentioned above.

Insects serve as a valuable source of proteins, lipids, minerals, vitamins, and energy and can be a promising alternative for both human consumption and animal feed [4]. When comparing edible insects to traditional livestock rearing, they offer several advantages, including the ability to consume low-value and waste materials [5], reduced environmental impact, high feed conversion rates, higher fecundity with a short life span, less space and water for rearing, and significantly lower emissions of greenhouse gases and ammonia [4,6,7].

Entomophagy, the practice of eating insects, traces back to ancient times and is a practice referenced in various religious texts, including Christian, Jewish, and Islamic literature [8]. Between 1600 and 2300 species of edible insects have been documented globally. These insects are consumed as both food and medicine by approximately two billion people from around 3000 ethnic groups over 113 countries, primarily situated in Africa, Asia, and Latin America [8,9].

The black soldier fly (BSF) (*Hermetia illucens* L., Diptera: Stratiomyidae) is a non-pest, adaptable, saprophagous insect native to the tropical and subtropical climates of the Americas, and it has been globally distributed across similar climates [10,11,12]. Black soldier fly larvae (BSFL) demonstrate a high bioconversion rate and possess great potential for large-scale production among insects, making them an economically advantageous option as feed [11,13]. They contain high amounts of protein with a balanced profile of essential amino acids and are a good source of lipids, including nutritionally valuable fatty acids [14]. Recently, BSFL meal has been successfully utilized as a part of feed for poultry [15], swine [16], and fish [17]. 

Food loss and waste pose significant challenges to global food security, economic sustainability, and environmental health [18]. As the global population continues to rise, so does the generation of waste and by-products, with approximately 1.3 billion tonnes generated annually today. Effective and urgent management of these waste streams is crucial [19]. Approximately one-third of the edible parts of food produced for human consumption worldwide are lost or wasted, estimated at around one trillion USD [18]. University canteens are among the places where a lot of food is wasted. The disposal of leftover plates leads to the wastage of significant quantities of diverse nutritional compounds derived from both plant and animal sources [20].

BSFL can be fed low-value organic waste streams and provide rich larval biomass and a good source of protein and oil [11]. Depending on the substrate they are fed with, the larvae’s protein and oil content can reach up to 53% and 41% on a dry matter basis, respectively [6]. Moreover, BSFL appear to be well adapted and capable of tolerating the presence of various pollutant substances, including the residues of pesticides, heavy metals, and mycotoxins, within their diets [10,21]. It seems that BSFL do not accumulate contaminants above the European feed legislation limits, except for certain metals (i.e., cadmium, lead, and zinc). However, there are still concerns about the other chemical compounds that require extensive research [10].

Sugarcane (*Saccharum officinarum*) is primarily cultivated globally for sugar and ethanol production in tropical and subtropical regions. In 2020, it reached a harvest volume of approximately 1.87 billion tonnes, making it the most-produced crop worldwide. However, sugarcane processing and its by-products can have adverse environmental impacts [22]. Iranian sugarcane is exclusively cultivated in Khuzestan province, located in the southwest. The annual sugarcane production in this region amounts to approximately eight million tonnes, covering an area of more than 100,000 ha [23]. Typical by-products of the sugarcane production industry include bagasse, molasses, press mud, vinasse, filter cake, cane trash, and bagacillo [22]. Bagasse is a fibrous residue left after the extraction of the sugar-bearing juice from sugarcane stalks. It is a valuable by-product of sugarcane processing and is commonly used as a biofuel, as animal feed, and in manufacturing paper and various building materials [22]. Vinasse, another by-product of sugarcane, is a dark liquid residue, abundant in organic matter and nutrients, generated during the distillation of sugarcane alcohol. However, its elevated levels of pollutants restrict its consumption, and its disposal poses significant environmental challenges [24]. Another common by-product of sugarcane, molasses, is composed of 45–55% sucrose, 20–30% glucose and fructose, and 10–20% water and is mainly used for animal feed, fermentation, production of ethanol, and other chemicals [22]. The objective of the present study was to compare the suitability of some sugarcane by-products, including bagasse, vinasse, and molasses, with accessible food waste like university canteen leftovers and carrot pomace for feeding BSFL. We tested their effects on the growth rate and chemical composition of BSFL.

## 2. Materials and Methods

### 2.1. Black Soldier Fly Colony

Black soldier fly eggs were collected from a colony established in September 2022 and maintained in the Agricultural Sciences and Natural Resources University of Khuzestan Entomology laboratory in Mollasani, Khuzestan, Iran. The source population of neonate larvae (3–4-day-old) was bought from the company Larviran in Mashhad, Iran. The insects were reared on kitchen waste for two generations before the tests. Egg traps made of triple-layer corrugated cardboard (5 × 3 cm) were used to collect eggs from the colony. The eggs used in this study were <6 h old [25] As the eggs came from multiple traps, first, they were homogenized by mixing them in a small plastic cup using a fine, dampened paintbrush. Then, three grams of eggs were weighed using a laboratory balance (A&D, EK600H, Tokyo, Japan) and introduced into 500 mL plastic containers containing a mixture of 50 g wheat bran and 40 g flour at 70% moisture. The containers were covered with lids, and ventilation holes were made to ensure proper airflow. The eggs were kept in the laboratory at 28.0 ± 1.0 °C and 70.0% relative humidity and for a photoperiod of 16:8 (L:D) h until hatching. Subsequently, the neonates were maintained under the same conditions for five days. 

### 2.2. Larval Feeding Experiments 

A randomized design was used in this study. The experiment used sterile plastic containers (23 × 13 × 10 cm) for larvae-rearing purposes. The containers were covered with a fine mesh fastened with a rubber band to prevent larval escape and probable contamination, and they were stored in the same environmental condition described previously. Substrates were weighted and distributed to the feeding containers using a uniform feeding rate of 100 mg/larvae/day [26]. About 1400 five-day-old larvae were evenly spread on the substrate in each container. The types of substrate used in these experiments are explained in detail in the next section. The amount of substrate was divided into two portions for each container and added in quantities shown in Table 1 each week. Some dry wheat bran was used around the premier of diets to prevent larvae from escaping. Three replicates were considered for each treatment.

### 2.3. Feeding Substrates

In this study, seven different substrates were used to rear BSFL. The substrate selection was based on the available food wastes and by-products. These substrates included the following: (1) carrot pomace (C); (2) carrot pomace + leftover bread (CB) (50/50); (3) bagasse + vinasse (BV); (4) bagasse + molasses (BM); (5) bagasse + vinasse + molasses (BVM); (6) a mixture of all above treatments (MX) including carrot pomace + leftover bread + bagasse + vinasse + molasses; and (7) university canteen leftovers (UCLs). The 5-day-old larvae were fed in two stages. The first portion of food was added on the first day of testing, and the remainder was added after one week (day 8). The quantities of different substrates in each treatment are shown in Table 1. Carrot pomace is a common waste in juice shops in Iran and was sourced from a local juice shop in Ahvaz, located in Khuzestan province, Iran, and bagasse, vinasse, and molasses are by-products of sugarcane industries in the Khuzestan province and were obtained from the Sugar Cane Plantation and Industry Company Dehkhoda, located in Ahvaz, Khuzestan, Iran, in November 2022. Due to the significant decrease in larval growth rate in the bagasse + molasses (BM) and bagasse + vinasse + molasses (BVM) treatments (Table 2), both treatments were excluded from chemical analyses and fatty acid profile tests. Bread is a staple in daily meals across the Middle East, and in Iran, leftover bread is often repurposed as livestock feed.

Leftover food from the plates was collected from the student canteen at the Agricultural Sciences and Natural Resources University of Khuzestan, Mollasani, Khuzestan, Iran. This comprised a mixture of vegetables, rice, and a variety of meats that were subsequently discarded by the canteen staff after lunch hour.

### 2.4. Larval Development

Larval weight and length were recorded in all replicates across treatments, starting from the first day after introducing larvae to various substrates. Ten larvae were sampled randomly from each container every other day from the first day of experiments. Considering the three replications for each diet, 30 larvae were collected from each treatment. Considering all six treatments, 180 larvae (30/treatment) were collected from containers each sampling day.

The larvae were killed using hot water (approximately 80 °C) for 10 s. The collected larvae from each replicate were then transferred to a Petri dish and weighed on an analytical balance (A&D, EK600H, Tokyo, Japan). The larval weight and length were measured within an hour of the killing process. The size of individual larvae was measured using a ruler. The tests were continued for two weeks. Then, the larvae were separated from the substrates, and all the larvae from each treatment were killed using hot water for 30 s for further analysis.

### 2.5. Proximate Composition

All of the larvae from each replication were collected separately to examine the chemical composition. Then, they were dried in an oven (Binder WTC, Tutlingen, Germany) at 50 °C until reaching a constant weight according to the AOAC official method (925.40). The dried larvae were ground into a fine powder using a 643 MX mill (Moulinex, Barcelona, Spain), passed through a 35-mesh sieve, and finally kept in zipper-lock polyethylene bags at −18 °C until analysis. The dry matter content was estimated by weighing the ground samples after oven-drying. The organic nitrogen amount was measured using the Kjeldahl method (ISO 5983-1, 2005) and converted to crude protein content by multiplication with a factor of 6.25. The ash content was evaluated by igniting samples at 550 °C in a muffle furnace (Fan Azma Gostar, model FG, Nazarabad, Alborz, Iran) for 6 h. The total oil content of samples was extracted with a 6-unit Soxhlet apparatus (Bakhshi Laboratory Industries, Tehran, Iran) using n-hexane as a solvent. Hexane evaporation was carried out under vacuum conditions to avoid oil oxidation during the extraction. The iron (Fe) and copper (Zn) concentrations were analyzed using an atomic absorption spectrometer (Contra 300, Analytik Jena, Jena, Germany). The flame photometer (Elico CL-361) was utilized to estimate the sodium (Na) and potassium (K) ions in the samples.

### 2.6. Fatty Acid Profile

The fatty acids of the samples were extracted and analyzed according to the method previously described, with slight changes [27]. In brief, approximately 50 mg of extracted oil from samples was saponified with 100 μL dichloromethane and 1 mL methanolic NaOH solution by refluxing for 10 min at 90 °C. Next, 1 mL of BF3-methanol (14%) was added to the solution and boiled in a water bath for 10 min. Fatty acid methyl esters (FAMEs) were extracted from a salt-saturated mixture with adding 600 μL n-hexane. Then, the organic layer was separated, and for quantitative analysis, 1 μL of it was injected into a Varian CP-3800 Gas Chromatograph (Varian, Walnut Creek, CA, USA) equipped with a flame ionization detector (FID) eluted with hydrogen gas at a flow rate of 29 mL/min, with a split ratio of 1:20. A long CP-SIL capillary column (100 m × 0.25 mm, 0.2 μm, Varian Inc.) was used to separating the FAMEs. The injector and detector were held at 300 °C and 280 °C, respectively. Analysis was carried out using nitrogen as the carrier gas. The temperature of the oven column was set at 70 °C and held for 1 min and then increased at 5 °C/min to 250 °C and held for 20 min. The methyl ester peaks were identified by their retention times and by comparing them with FAME standards from Sigma-Aldrich, which were analyzed under the same conditions. The analysis of FAMEs was run in triplicate (*n* = 3). 

### 2.7. Statistical Analysis

The statistical analysis of data was performed using IBM SPSS Statistics v. 20.0 for Windows. The normality of data distribution was evaluated using the Shapiro–Wilk test, revealing no significant deviations from normality (*p* > 0.05). Additionally, the homogeneity of variances was confirmed through Levene’s test (*p* > 0.05), indicating that the variances across treatment groups were homogeneous. The average larval length and weight were calculated by Excel software, and the data obtained from the final sampling date were compared among treatments using one-way ANOVA (α = 0.05), with Tukey’s test as a post hoc test. The one-way ANOVA (α = 0.05) and Tukey’s test were also used to compare the crude protein, oil, ash, dry matter, mineral, and fatty acid contents of the larvae among substrates. 

## 3. Results

### 3.1. Larval Development

**Figure 1:**
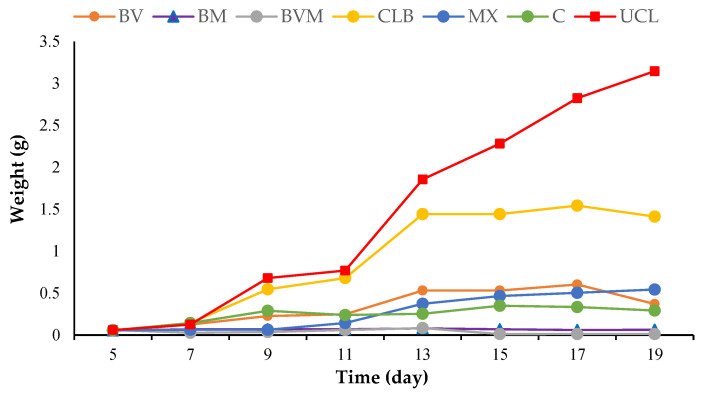
The mean weight of the Black soldier fly larvae reared on different substrates for 14 days from day 5 to 19. UCL = university canteen leftover, CLB = carrot pomace + leftover bread, BV = bagasse + vinasse, C = carrot pomace, MX = mixture of bagasse + molasses + vinasse + carrot + bread, BVM = bagasse + vinasse + molasses, BM = bagasse + molasses.

The growth rate of black soldier fly larvae on different diets was monitored from day 2 to day 14. The weight of the larvae on the last sampling date was significantly affected by the ingredients of the diets (F: 155.08; df = 6.20; *p* < 0.0001) (Table 2 and Figure 1). The total larval weight was the highest in the UCLs (3.15g) and the lowest in the BM diet (0.06 g). The CLB diet had the most influence on the larval weight (1.41 g) after the university canteen leftover diet. However, the other diets had no significant differences (Table 2).

The diet substrate also affected larval length during the 14 days of monitoring (F = 50.261, df = 6.69; *p* < 0.0001). The longest larvae were recorded in the UCL (17.00 mm) and CLB diets (14.60 mm) (Table 2). The BSF larvae showed the shortest length in the BM and BVM diets, at 3.60 and 6.00 mm, respectively. These results showed that although the larvae were longer on the C (11.70 mm) and the MX (10.40 mm) diets than on the BM and BVM diets, their length significantly decreased compared to the UCL, CLB, and BV diets (Table 2).

### 3.2. Proximate Composition

The content of crude protein, crude oil, dry matter, ash, sodium (Na), potassium (K), iron (Fe), and zinc (Zn) in the samples was measured, and the results are presented in Table 3.

The substrates significantly affected the larvae’s chemical composition and elemental content (*p* < 0.05). Larval crude protein varied from 32.6 to 38.9% DM and significantly differed among the samples. The larvae reared on the UCLs had a protein content similar to CLB, with values of 38.9% and 38.7%, respectively, but their protein content was higher than those reared on other substrates (*p* < 0.05). The crude oil content ranged between 28.1 and 39.06%, showing significant differences among the treatments, with the highest oil content found in larvae fed with UCLs and the lowest in larvae fed with C. Meanwhile, the larvae grown on the BV substrate showed the highest amount of ash with values of 28.9%, while those fed with UCLs exhibited the lowest amount (5.7%). The dry matter of the samples ranged from 24.90 to 28.03%. The various substrates were associated with a dry matter (*p* < 0.05), and the larvae reared on carrot pomace and BV had the lowest and highest percentage.

The results also demonstrated that the type of substrate the larvae fed on significantly impacted the measured Na, K, Fe, and Zn (*p* < 0.05). Among the minerals measured in this study, potassium (K) was the most dominant mineral in BSFL across all substrate types, with values ranging from 30.5 to 120.5 ppm, followed by Na and Fe, with concentrations ranging from 15 to 18.5 and 1.15 to 1.23–66 ppm, respectively. However, the level of Zn was observed to be lower than the other minerals. The larvae grown on UCLs contained the highest amounts of Na (18.5 ppm) and Fe (1.23 ppm), whereas the larvae that fed on C showed the highest levels of K (120.5 ppm) and Zn (1.11 ppm). Interestingly, the lowest values of the studied elements were observed in the larvae reared on the MX substrate.

### 3.3. Fatty Acid Profile

The fatty acid profile of larvae grown on different substrates is presented in Table 4. As indicated in the table, the larval feeding diet significantly influenced the amount of their fatty acids (*p* < 0.05). The results of the fatty acid analysis of the samples revealed that the oil extracted from the larvae grown on different substrates contained significantly higher levels of saturated fatty acids (SFAs) compared to unsaturated fatty acids (UFAs). The oil obtained from all the investigated larvae included between 66.39 and 75.54% saturated fatty acids.

Lauric acid (C12:0) was identified as the most abundant fatty acid in the BSFL across all the substrates, with levels ranging from 40.40% in BV to 48.12% in UCLs. The highest amount of lauric acid was observed in the larvae fed with UCLs. After lauric acid, significant amounts of oleic acid (16.20–18.90%) and palmitic acid (16.01–17.56%) were observed in all the samples. The larvae reared on BV and MIX substrates had the highest amount of oleic and palmitic acid, respectively.

Palmitoleic, oleic, linoleic, and linolenic acids were identified as unsaturated fatty acids in the oil of larvae grown on different substrates, with the total amount of unsaturated fatty acids ranging between 25.15% and 33.21%. The lowest and highest amounts of unsaturated fatty acids were observed in larvae fed with UCLs and BV, respectively.

## 4. Discussion

With global sugar production expected to rise in the next decade, especially in Brazil, India, and Thailand, a significant increase in sugarcane by-products is anticipated [28]. While these by-products are already utilized in various industries, their potential as sustainable feed sources for BSFL have not been fully explored. This study compared the growth and composition of BSFL reared on sugarcane by-products and other substrates, offering new insights into their viability as a novel resource in insect farming. Such findings could promote more sustainable waste management practices and contribute to circular economy efforts.

Our findings showed that BSFL could feed on different kinds of tested substrates. However, they grew well on UCLs and CBL, producing nutrient-rich biomass. Regarding nutrient composition, significant differences in crude oil, crude protein, dry matter, and ash levels were observed among the BSFL fed with different substrates. Previous studies have demonstrated the impact of various biowastes and by-products used as rearing substrates on the chemical compound content of reared BSFL [11,16,29,30,31]. Although sugarcane molasses is widely used as an energy source to feed animals, the BSFL did not grow well in substrates containing molasses. This might be due to the high amounts of sugar, including sucrose, glucose, and fructose, which create an adhesive environment that can prevent the normal movement and growth of the larvae. Therefore, because of the small size, we excluded the larvae obtained from the BVM and BM treatments from further evaluation. 

In this study, the UCL and CBL treatments produced longer and heavier larvae over the 14-day study period compared to other treatments, which means a shorter development time for the larvae before turning into prepupae. The UCLs mainly contained different protein sources, including meat, rice, and less bread. CBL was also composed of an equal amount of carrot pomace and leftover bread. Previous studies also showed faster developmental time and larger sizes for larvae fed with protein-rich substrates [7,32]. In our study, the BSFL raised on BV substrates ranked second in terms of length and weight, following the UCL and CBL treatments. Studies have shown that vinasses are rich in protein, especially non-essential amino acids [33]. Carrot pomace content mainly depends on the variety of carrot, but in general, it had a high content of fiber (20.09–33.34%), carbohydrate (46.55–58.95%), ash (5.29–5.89%), and protein (6.87–9.14%) [34,35]. Both the C and BV substrates had higher moisture levels, resulting in smaller larvae compared to those on the UCL and CBL substrates. The moisture content of the substrate significantly impacts the growth, development, and survival of BSFL. Optimal moisture levels typically range between 50% and 70%, as moisture influences the texture of the substrate, which in turn affects larval movement, feeding efficiency, and overall growth [36]. Lower moisture levels (30%) tend to reduce larval weight and survival rates, while excessively high moisture content (above 80%) can lead to poor larval growth due to substrate compaction and reduced oxygen availability [36,37]. On the other hand, although the MX included bread leftovers and vinasse, the larvae developed slower than the other protein-based substrates. It can be explained by molasses in the MX substrates, which, as mentioned before, may restrict the growth of the larvae. 

The crude protein and crude fat content of the BSF larvae ranged from 32.6 to 38.9%, and 28.1 to 39.06% on a dry matter basis, respectively. These values are consistent with previous studies, such as those by Spranghers [11] , who reported 40% crude protein and 33% crude fat in larvae reared on substrates like chicken feed and vegetable waste, and Chia et al. [38], who observed 29.9–45.7% crude protein and 9.5–49% crude fat in larvae grown on dried brewers’ spent grains. Nyakeri et al. [31] also reported an average of 38.9% crude protein and 32.6% crude fat in wild BSFL reared on various organic waste substrates. In this study, larvae reared on BV substrates had the least oil (28.9%) compared to other treatments. This might be related to the low content of carbohydrate in the BV substrates as the BSFL convert the carbohydrates into lipids, which the adults will then use as energy storage molecules [31]. 

The ash content levels of the BSFL ranged from 5.7 to 28.9%, which align with the ranges reported in the literature [38,39]. On the other hand, dry matter varied between 24.90 and 28.03%, which is quite high and means the drying process of larvae would be easy and cost-effective. The variation in ash content levels highlights the influence of substrate composition on the nutritional profile of BSF larvae. The ash content is attributed to the quantity of various minerals such as iron, calcium, phosphorus, copper, zinc, etc. [40]. Our study discovered the highest percentage of ash in the BV treatment. In addition to ash, the BV treatment showed a high protein content, which might be a clue to the high nutritional value of the BSFL reared on this substrate [38].

Our findings also clearly indicated that the mineral content of BSF larvae is influenced by the growth substrate. Among the minerals studied here, potassium (K) emerged as the dominant mineral in all samples, with levels ranging from 30.5 to 120.5 mg/kg, significantly higher than sodium (Na) and iron (Fe), which were in the ranges of 15–18.5 mg/kg and 1.15–1.23 mg/kg, respectively. This is consistent with Adebayo’s study, which also identified potassium as the predominant mineral in larvae reared on substrates like chicken feed and food waste [29]. While potassium was the most abundant mineral observed, previous research has indicated that calcium and phosphorus are the significant minerals found in BSF larvae, depending on the substrates used [11,38]. At harvest time, BSFL may still have some of their diet in their gastrointestinal tracts, which can significantly influence the results. This remaining food may impact the type and amount of minerals detected in BSFL. Investigating the substrate composition and calcium and phosphorous content of BSFL reared on UCLs and other substrates can be considered a subject of future studies. 

The fatty acid composition of BSFL is significantly influenced by the substrates on which they are reared. In our study, we observed distinct variations in the levels of various fatty acids, highlighting the impact of diet on the biochemical makeup of the larvae. Based on the fatty acid profile data, saturated fatty acids were found to be the leading group of fatty acids in all the BSFL, which is expected given the high levels of lauric and palmitic acids, and this is in line with findings from several earlier studies [11,19,41,42,43]. Irrespective of diet, lauric acid was the most abundant fatty acid in the BSFL. Previous studies have also identified lauric acid as the predominant fatty acid in BSFL, with levels of up to 63% of total fatty acids. This reveals that BSFL synthesize lauric acid from the carbohydrates in the substrate [11]. Lauric acid is known for its antimicrobial properties and is valuable in various industrial applications, including cosmetics and food products [44]. Following lauric acid, oleic and palmitic acids constitute a high percentage of the fatty acids detected in larvae. The concentrations of these fatty acids varied depending on the substrate. Oleic acid and palmitic acid are essential for insects’ energy storage and membrane structure. Oleic acid is also a precursor to linoleic acid, which is crucial for insects’ metabolism [45]. The current results agree with the research of others who used different sources to feed larvae and reported lauric acid, palmitic acid, and oleic acid as the predominant fatty acids in BSFL [11,19,42].

The balance between SFAs and UFAs is essential for maintaining the structural integrity of cell membranes and ensuring proper physiological function in insects [46]. The presence of both SFAs and UFAs in considerable amounts indicates that BSFL can effectively incorporate and synthesize these fatty acids to meet their metabolic needs. BSFL can produce these fatty acids through biosynthetic pathways as well as by accumulating them from their diet [43]. The composition of substrates influences the fatty acid profiles of BSFL; particularly, the presence of carbohydrates can lead to the de novo biosynthesis of saturated fatty acids [47]. Carbohydrates are metabolized into acetyl-CoA, a precursor for fatty acid synthesis. This process is facilitated by enzymes such as acetyl-CoA carboxylase and fatty acid synthase [43,47]. These enzymes play a crucial role in the conversion of dietary carbohydrates into SFAs, highlighting the metabolic flexibility of BSFL in adapting to various dietary inputs [47]. This indicates that larvae might alter their fatty acid profiles in response to changes in the nutrient content of the substrate [41,42].

## 5. Conclusions

This study demonstrates that sugarcane by-products can be effectively used as rearing substrates for BSFL, offering a sustainable solution for agricultural waste. Among different sugarcane by-products used as treatments, BV was the most appropriate for BSFL development. The larvae grew better in BV than in other sugarcane by-product combinations. The larvae’s growth and nutrient composition make them a promising alternative protein source for animal feed, potentially reducing reliance on conventional protein sources like soybean meal. By utilizing waste streams like sugarcane by-products, BSFL farming can contribute to more sustainable animal feed production, aligning with circular economy principles and promoting environmental sustainability. Future research should continue to explore the impact of adding vinasse to different substrates on BSFL’s detailed nutrient and mineral profiles. We also recommend using vinasse on an industrial scale to compare their efficiency with our small-scale study. 

## Figures and Tables

**Table 1:** Substrate quantities by treatment and feeding stage.

Treatment	Day 1	Day 8
Carrot pomace (C)	700 g	700 g
Carrot pomace + leftover bread * (CLB)	700 g	700 g
Bagasse + vinasse (BV)	130 g + 650 mL	25 g + 200 mL
Bagasse + molasses ** (BM)	130 g + 650 mL	25 g + 200 mL
Bagasse + vinasse + molasses (BVM)	100 g + 300 mL + 300 mL	50 g + 100 mL + 100 mL
Mixture of bagasse + molasses + vinasse + carrot + bread (MX)	90 g + 120 mL + 120 mL + 175 g + 175 g	10 g + 50 mL + 50 mL + 175 g + 175 g
University canteen leftover (UCL)	700 g	700 g

* The bread used in this study was soaked in water before being added to the substrates. ** All molasses treatments in this experiment included molasses + water (1:1 *v*/*v*).

**Table 2:** The length and weight (mean ± SE) of BSF larvae reared on various substrates.

	Diets *						
	UCL	CLB	BV	C	MX	BVM	BM
**Length (mm)**	17.00 ± 0.61 a **	14.60 ± 0.52 a	11.20 ± 1.00 b	11.70 ± 0.72 b	10.40 ± 0.83 b	6.00 ± 0.26 c	3.60 ± 0.22 c
**Weight (g)**	3.15 ± 0.15 a	1.41 ± 0.13 b	0.64 ± 0.54 c	0.29 ± 0.05 cd	0.54 ± 0.72 c	0.14 ± 0.00 d	0.06 ± 0.00 d

* UCL = university canteen leftover, CLB = carrot pomace + leftover bread, BV = bagasse + vinasse, C = carrot pomace, MX = mixture of bagasse + molasses + vinasse + carrot + bread, BVM = bagasse + vinasse + molasses, BM = bagasse + molasses. ** Means within the same row followed by the same letter are not significantly different (*p* < 0.05, Tukey’s test).

**Table 3:** Proximate composition and some mineral content of BSF larvae reared on various substrates.

Treatments *	Protein%	Oil%	Ash%	Dry matter%	Na (ppm)	K (ppm)	Fe (ppm)	Zn (ppm)
**UCL**	38.9 ± 0.66 a**	39.06 ± 0.59 a	5.7 ± 0.20 e	27.06 ± 0.13 b	18.5 ± 0.28 a	86.2 ± 0.41 c	1.23 ± 0.23 a	0.31 ± 0.00 d
**BV**	37.3 ± 0.36 b	28.1 ± 0.20 e	28.9 ± 1.05 a	28.03 ± 0.06 a	17.8 ± 0.21 ab	96.1 ± 0.20 b	1.18 ± 0.01 b	0.77 ± 0.02 c
**MX**	35.2 ± 0.43 c	34.6 ± 0.72 c	22.0 ± 0.57 b	27.41 ± 0.19 b	15.0 ± 0.57 c	20.5 ± 0.28 d	1.15 ± 0.01 b	0.73 ± 0.02 c
**C**	32.6 ± 0.21 d	31.1 ± 0.37 d	14.9 ± 0.55 c	24.90 ± 0.30 c	16.8 ± 0.15 b	120.0 ± 2.30 a	1.17 ± 0.01 b	1.11 ± 0.04 a
**CLB**	38.7 ± 0.43 a	36.9 ± 0.49 b	7.8 ± 0.30 d	27.20 ± 0.17 b	17.2 ± 0.41 b	94.0 ± 1.15 b	1.17 ± 0.01 b	0.88 ± 0.04 b

Number of replicates: 3; the minerals are represented on a dry matter basis. * UCL = university canteen leftover, CLB = carrot pomace + leftover bread, BV = bagasse + vinasse, C = carrot pomace, MX = mixture of bagasse + molasses + vinasse + carrot + bread. ** Means within the same column followed by the same letter are not significantly different (*p* < 0.05, Tukey’s test).

**Table 4:** Fatty acid composition (expressed as a percentage of total fatty acids) of BSF larvae reared on various substrates.

		Treatments				
Fatty Acid	RT (min)	UCL	BV	MIX	C	CLB
Caprylic acid (C8:0)	7.256	1.10 ± 0.24 a	1.23 ± 0.14 a	1.16 ± 0.33 a	1.16 ± 0.23 a	1.16 ± 0.1 6 a
Capric acid (C10:0)	9.514	1.00 ± 0.14 b	1.54 ± 0.21 a	1.15 ± 0.18 b	1.15 ± 0.14 b	1.14 ± 0.22 b
Lauric acid (C12:0)	12.41	48.12 ± 0.15 a	40.40 ± 0.19 d	43.11 ± 0.20 c	44.85 ± 0.11 b	44.01 ± 0.23 b
Myristic acid (C14:0)	15.029	6.25 ± 0.2 a	5.21 ± 0.24 b	4.42 ± 0.23 c	3.43 ± 0.21 d	6.42 ± 0.28 a
Palmitic acid (C16:0)	15.567	16.32 ± 0.23 b	16.12 ± 0.41 b	17.56 ± 0.36 a	16.56 ± 0.19 b	16.01 ± 0.25 b
Palmitoleic acid (C16:1)	16.061	2.61 ± 0.16 c	3.21 ± 0.12 a	2.99 ± 0.15 ab	2.88 ± 0.08 b	3.03 ± 0.11 ab
Stearic acid (C18:0)	29.412	1.95 ± 0.18 a	1.89 ± 0.23 a	1.95 ± 0.17 a	1.95 ± 0.18 a	1.95 ± 0.24 a
Oleic acid (C18:1n-9)	31.515	18.21 ± 0.23 b	18.90 ± 0.15 a	17.50 ± 0.28 c	18.20 ± 0.23 b	16.20 ± 0.21 d
Linoleic acid (C18:2)	33.021	3.10 ± 0.15 d	7.40 ± 0.23 b	6.20 ± 0.25 c	7.35 ± 0.27 b	8.30 ± 0.25 a
Linolenic acid (C18:3)	34.03	1.23 ± 0.08 d	3.70 ± 0.19 a	3.10 ± 0.22 b	2.23 ± 0.32 c	1.23 ± 0.19 d
SFAs		74.54 ± 0.25 a	66.39 ± 0.19 d	69.35 ± 0.03 c	69.10 ± 0.49 c	70.69 ± 0.57 b
UFAs		25.15 ± 0.47 d	33.21 ± 0.19 a	29.79 ± 0.41 b	30.66 ± 0.92 b	28.76 ± 1.16 c
UFAs/SFAs		0.33 ± 0.05 d	0.50 ± 0.07 a	0.43 ± 0.05 b	0.44 ± 0.03 b	0.40 ± 0.07 c

Number of replicates: 3. UCL = university canteen leftover, CLB = carrot pomace + leftover bread, BV = bagasse + vinasse, C = carrot pomace, MX = mixture of bagasse + molasses + vinasse + carrot + bread. Means within the same column followed by the same letter are not significantly different (*p* < 0.05, Tukey’s test).

## Data Availability

The data presented in this study are all included on the manuscript.
